# Food insecurity in Brazil by household arrangements and characteristics between 2004 and 2022

**DOI:** 10.1590/0102-311XEN168823

**Published:** 2024-06-14

**Authors:** André Braz Golgher

**Affiliations:** 1 Universidade Federal de Minas Gerais, Belo Horizonte, Brasil.

**Keywords:** Family Characteristics, Economic Recession, COVID-19, Pandemics, Food Insecurity, Características da Família, Recessão Econômica, COVID-19, Pandemias, Insegurança Alimentar, Composición Familiar, Recesión Económica, COVID-19, Pandemias, Inseguridad Alimentaria

## Abstract

Although food insecurity presents a decreasing trend worldwide, some regions recently observed an increase in hunger levels. Such was the case in Brazil between 2014 and 2018, during and after the great Brazilian recession, and between 2020 and 2021, during the COVID-19 pandemic. This paper describes the evolution of food insecurity in Brazil between 2004 and 2022 using *Brazilian National Household Sample Survey* (PNAD), *Brazilian Household Budget Survey* (POF) and Continuous PNAD. Households were classified in 20 types of arrangements, and the most vulnerable living arrangements between 2004 and 2018 were identified by multinomial logistic models. Overall, households headed by women (single blacks, whites or in couples) with or without children were the most prone to food insecurity. As for the evolution of food insecurity in Brazil between 2018 and 2022, logistic models were applied to estimate moderate and severe food insecurity levels among the 20 household types. Additionally, effects of the emergency aid and idiosyncrasies of the COVID-19 pandemic were estimated.

## Resumo

A tendência geral de insegurança alimentar no mundo é decrescente. No entanto, algumas regiões observaram um recente aumento nos níveis de fome. Isso ocorreu no Brasil de 2014 a 2018, durante e após a grande recessão brasileira, e de 2020 a 2021, durante a pandemia da COVID-19. Este artigo descreve a evolução da insegurança alimentar no Brasil de 2004 a 2022 usando dados obtidos através das seguintes pesquisas: *Pesquisa Nacional por Amostra de Domicílios* (PNAD), *Pesquisa de Orçamentos Familiares* (POF) e PNAD Contínua. Os domicílios foram classificados em 20 tipos de arranjo domiciliar. Inicialmente, modelos logísticos multinomiais foram aplicados para identificar os arranjos domiciliares mais vulneráveis de 2004 a 2018. Em geral, os domicílios chefiados por mulheres (negras, brancas ou casais) com ou sem filhos foram os mais propensos à insegurança alimentar. Além disso, foi abordada a evolução da insegurança alimentar no Brasil entre os arranjos domiciliares de 2018 a 2022. Modelos logísticos foram aplicados para estimar os níveis de insegurança alimentar moderada e grave para os 20 tipos de arranjos domiciliar. Além disso, foram estimados os efeitos do auxílio emergencial e as peculiaridades da pandemia da COVID-19.

## Resumen

La tendencia general de la inseguridad alimentaria está disminuyendo en el mundo. Sin embargo, algunas regiones han registrado un aumento reciente en los niveles de hambre. Esto había ocurrido en Brasil en el período de 2014 a 2018, durante y después de la gran recesión brasileña, y de 2020 a 2021, durante la pandemia del COVID-19. Este artículo describe la evolución de la inseguridad alimentaria en Brasil de 2004 a 2022 a partir de datos obtenidos de las siguientes encuestas: *Encuesta Nacional por Muestra de Domicilios* (PNAD), *Encuesta de Presupuesto de Familiares* (POF) y PNAD Continua. Se clasificaron los hogares en 20 tipos de arreglos domésticos. Inicialmente, se aplicaron modelos logísticos multinomiales para identificar los arreglos domésticos más vulnerables en el período de 2004 a 2018. En general, los hogares que tienen a mujeres (negras, blancas o en pareja) con o sin hijos como jefas del hogar fueron los más propensos a la inseguridad alimentaria. Además, se abordó la evolución de la inseguridad alimentaria en Brasil en los arreglos domésticos para el período de 2018 a 2022. Se aplicaron modelos logísticos para estimar los niveles de inseguridad alimentaria moderada y severa para los 20 tipos de arreglos domésticos. Además, se estimaron los efectos de la ayuda de emergencia y las peculiaridades de la pandemia del COVID-19.

## Introduction

As per the Sustainable Development Agenda vision of a world without hunger, food insecurity decreased in most parts of the world between 2005 and 2014. However, undernourishment levels stabilized between 2014 and 2019, whereas food insecurity actually increased in middle-income countries in Latin America, including Brazil ^1^, which saw low economic growth in the period ^2^.

Brazil’s gross domestic product (GDP) grew over 2% annually between 2001 and 2013 ^2^. This trend changed afterwards due to the great Brazilian recession from 2014 to 2016, when GDP decreased close to 4% yearly, constituting the most marked drop in the country’s economic activity between the end of the Second World War and the COVID-19 pandemic ^3^. Between 2017 and the COVID-19 pandemic onset, the Brazilian economy grew slightly, indicating a timid recovery. GDP dropped to 3.3% between 2019 and 2020 due to the pandemic, and then saw a recovery in the following years ^4^.

Economic crises like the great Brazilian recession and the pandemic one challenge access to food and to essential social services, potentially increasing food insecurity ^1^. In such periods, households adopt coping strategies to overcome the increased hardships, reducing overall food intake and switching to less preferred types of food. Moreover, financial and food crises most likely affect the same population strata, such as minorities, the poor and vulnerable female-headed households ^5^.

Some studies ^6^ ^,^ ^7^ ^,^ ^8^ ^,^ ^9^ have estimated food insecurity using indicators from the *Brazilian Food Insecurity Scale* (EBIA, acronym in Portuguese), considered the main food insecurity assessment tool in Brazil. Felker-Kantor & Wood ^6^ compared moderate and severe food insecurity levels for different household compositions using the *Brazilian National Household Sample Survey* (PNAD, acronym in Portuguese). The authors observed that households headed by women and those with young children and adolescents had higher levels of both food insecurity types, and verified that the presence of more adult females compared with more adult males, reduced food insecurity. This finding is consistent with women’s spending patterns which are more focused on the household than men’s ^10^.

Souza et al. ^7^ described changes in Brazilian food security levels between 2003 and 2017 using PNAD and data from the Gallup World Poll, concluding that despite significant advances between 2004 and 2013, Brazil suffered from a great deterioration afterwards. Moreover, households with lower per capita income, lower schooling levels and more residents tended to present higher food insecurity levels. Conversely, households with older individuals tended to be more food secure.

Brazilian Research Network on Food Sovereignty and Security (Rede PENSSAN, acronym in Portuguese) ^8^ analyzed associations between the COVID-19 pandemic and food insecurity in Brazil. VIGISAN I (*National Survey on Food Insecurity in the Context of the COVID-19 Pandemic in Brazil*), conducted at late 2020, found that 55.2% of the households faced food insecurity at different levels and 9% lived with severe food insecurity. VIGISAN II, conducted between late 2021 and early 2022, showed higher values: 58.7% and 15.5%, respectively.

Salles-Costa et al. ^9^ discussed the evolution of food insecurity in Brazil between 2004 and 2022, highlighting that food insecurity levels at the end of the period were worse than in the beginning, and attributed this increase partially to the dismantlement of public policies aimed at promoting food security. The Brazilian National Food and Nutritional Security System (SISAN, acronym in Portuguese) and programs strengthening small farmer’s productivity faced budget cuts in 2016. Despite food prices inflation, the municipal and state budgets per student allocated by the Brazilian National School Feeding Program (PNAE, acronym in Portuguese) was not nominally increased after 2017. Moreover, the Food and Nutrition Security National Council (Consea, acronym in Portuguese) was closed in 2019. Conversely, the emergency aid was implemented in July 2020 to mitigate negative effects of the COVID-19 pandemic on food insecurity ^11^ ^,^ ^12^.

Given this scenario, this paper described the food insecurity evolution in Brazil from 2004 to 2018 giving particular attention to household arrangements and characteristics. Multinomial logistic models were applied to PNAD and to the *Brazilian Household Budget Survey* (POF, acronym in Portuguese) databases to identify the most vulnerable living arrangements. Additionally, it estimated food insecurity by household type regarding specific pandemic effects and impacts of the emergency aid. A logistic model was designed using 2013 PNAD, which was then applied to estimate moderate/severe food insecurity for the different living arrangements using the 2018, 2020-2021 and 2022 Continuous PNAD. Compared to other studies dealing with Brazilian data, this paper innovates by focusing on living arrangements, proposing different main objectives and methodology, and by including the COVID-19 pandemic period.

## Methodology

### Databases

This research used the PNAD (2004 ^13^ and 2013 ^14^), POF (2017-2018 ^15^) and Continuous PNAD (2018 ^16^, 2020-2021 ^17^ and 2022 ^18^) databases, which are nationally representative ^9^. Households headed by Indigenous people were excluded as these may face a rather different food insecurity reality than other households in Brazil.

Between 2004 and 2013, PNAD was surveyed annually by the Brazilian Institute of Geography and Statistics (IBGE, acronym in Portuguese), except in 2010, a census year. It brings special supplements in specific years and food security was investigated in 2004, 2009 and 2013. Continuous PNAD replaced the traditional PNAD in 2016. Regarding POF, Conde et al. ^19^ estimated underweight and obesity trends among Brazilian adults using the 2002-2003 and 2008-2009 publications. This survey is also conducted by IBGE.

### EBIA

Food insecurity in Brazil is commonly measured using EBIA, 14-question instrument ^20^, all answered as yes or no. Positive answers are summed and households are classified into four categories: food secure, mild food insecurity, moderate food insecurity and severe food insecurity.

Reichenheim et al. ^21^ suggested that if grouping EBIA classes is desired, food secure and mild food insecurity could be analyzed as a single stratum. To designa food insecurity dummy, the authors suggest further merging the moderate and severe food insecurity categories.

Initial analysis used the traditional four-category EBIA. The first two categories were then grouped to form the dependent variable for the multinomial logistic models (secure/mild food insecurity; moderate food insecurit; severe food insecurit), and the last two were merged to create the dependent variable for the logistic model (secure/mild food insecurit; moderate/severe food insecurity).

### Household types

Initially, household heads were classified by skin color/ethnicity into two groups: white/Asian and black/mixed (henceforth named as white and black for simplicity). This type of grouping is commonly performed for Brazilian data ^22^. Moreover, household heads were classified as young adults if under 65 years old, and as old adults, if aged 65 and over. Individuals in the household of any age classified as son/daughter or stepson/daughter were categorized as children and other household residents as aggregates.

Households were classified into 20 types, all with at least 300 observations in each database, of which 16 were headed by someone younger than 65 years old. These living arrangements differ by the presence of children (yes; no), sex of the household head (male; female), skin color of the household head (white; black) and by the presence of a couple (yes; no).

The remaining four household types are headed by an old adult, and were classified by whether other people resided in the household besides the head or the couple (yes; no) and by the household head’s skin color (white; black). Offspring in these households are usually adults and were grouped with the aggregates. Presence or not of a couple and the head’s sex was not considered for classification to keep the number of types smaller and with more observations.

### Empirical strategy

The empirical strategy consisted of two models, each addressing one of the main research objectives. First, multinomial logistic models were applied to the PNAD and POF data to associate food insecurity evolution with household arrangements and characteristics between 2004 and 2018. Similar models were estimated for each year separately to verify possible different food insecurity trends between the living arrangements and to describe some of the main food insecurity-related household characteristics.

Second, logistic models were estimated using the 2013 PNAD, and the probability of moderate/severe food insecurity was estimated for each household using the 2018, 2020-2021 and 2022 Continuous PNAD. Simulations calculated the proportion of moderate/severe food insecurity households in each arrangement in three scenarios. The first simulation adjusted the 2018 moderate/severe food insecurity level to the actual level observed in this year to unveil the pandemic effects directly associated with labor market outcomes on food insecurity in 2020, 2021 and 2022. The second adjusted the 2020 moderate/severe food insecurity level to the actual level observed in this year to verify the effects of the 2020 emergency aid and to analyze short-term effects of the pandemic. Finally, the third simulation adjusted 2021 moderate/severe food insecurity levels to the actual level observed in this year to investigate the effects of the 2021 emergency aid.

### Other variables

In addition to household types, the multinomial logistic models included a set of explanatory variables, all available in PNAD (2004 and 2013) and in POF (2017-2018), associated with socioeconomics status level and food insecurity in the long term ^6^ ^,^ ^7^ ^,^ ^8^ ^,^ ^9^. Regarding socioeconomic levels, the variables consist of a categorical variable for household head formal schooling level (0 to 4 years; 5 to 8 years; 9 to 11 years; 12 years; 13 or more years), dummies (no; yes) for households with brick walls, proper sanitation, waste collection, with TV or refrigerator, and dwellers density per room (continuous). Labor market income was summed for all household residents and values were deflated. Since many households had zero labor market income, the value was summed to one before applying the natural logarithm of total labor market income. Importantly, variable refers to labor market-related total household income and not household income per capita. As for household location, models included a dummy for urban area (no; yes) and a categorical variable for state (27 categories).

However, some of those variables are not strictly associated with labor market short-term fluctuations related to economic and public health crisis. Variations in unemployment rates, relative proportion of precarious and poorly paid jobs and household income losses only partially explain the increase in severe food insecurity during the COVID-19 pandemic ^9^. A different set of explanatory variables, present in PNAD (2013) and Continuous PNAD (2018, 2020-2021 and 2022), is thus included in studies associating the pandemic with food insecurity. These consisted of labor market variables concerning the household head: a dummy for unemployed (no; yes), a categorical variable for labor market participation (no participation; not occupied or working less than 15 hours weekly; working from 15 to 39 hours weekly; full time worker). Moreover, food insecurity depends not only on household characteristics but also on the social environment surrounding them. One variable was created to address this point: the percentage of households with household heads outside the labor market, not occupied or working up to 15 hours weekly by state.

A dummy was created for non-labor market funds (zero; positive), using values available in the PNAD (2013) and 2018 Continuous PNAD. Values for 2020, 2021 and 2022 were imputed using logistic models based on 2018 data. Explanatory variables for this imputation are those associated with the Brazilian Income Transfer Program (*Bolsa Família*) and retirement, mostly already defined above.

The Federal emergency aid, varying between BRL 600 and BRL 1,200 depending on household composition, benefited close to 70 million individuals in 2020. It was suspended in early 2021 and then implemented again in the same year, with under 40 million people receiving approximately half of the previous amount ^11^ ^,^ ^12^. To estimate the effects of these policies, we calculated the expected probabilities of each household receiving this aid using a standard regression model with ordinary least squares (OLS) applied to 2018 PNAD. Total household income was imputed as the dependent variable, whereas the explanatory variables have already been defined above. Total number of individuals receiving the emergency aid was obtained with the expected probabilities according to the actual values for each year. The amount received by each household followed the household profile ^11^ ^,^ ^12^.

Finally, two associations were included in the models: one between non-labor market funds and the dummy for households headed by an old adult, representing differences between cash transfers and retirement; and another between weekly workload and household types, due to possible different participations in the labor market.

### Ethical aspects

All data used in this study is publicly available and freely downloaded.

## Results

### Descriptive statistics

Table 1 shows the household distribution by type for the period between 2004 and 2022. Its upper panel shows the results for the 20 arrangements, whereas the bottom panel synthetize the findings regarding five characteristics.

**Table 1:** Household distribution by type and by characteristics between 2004 and 2022.

Characteristics	2004 (%)	2013 (%)	2018 (%)	2022 (%)
Black women with children	6.6	7.8	7.9	8.6
Black women without children	0.9	1.5	1.9	2.2
Couple black women headed with children	1.8	7.0	9.2	14.1
Couple black women headed without children	0.2	1.1	1.4	2.4
Black men with children	0.7	0.9	0.8	1.0
Black men without children	1.1	1.7	2.0	3.2
Couple black men headed with children	30.1	24.8	22.6	16.3
Couple black men headed without children	2.5	3.9	3.7	3.1
White women with children	5.8	5.0	4.5	4.4
White women without children	1.2	1.3	1.5	1.8
Couple white women headed with children	1.5	4.9	5.5	9.0
Couple white women headed without children	0.3	1.0	1.3	2.0
White men with children	0.6	0.6	0.6	0.6
White men without children	1.2	1.4	1.5	2.4
Couple white men headed with children	30.1	19.2	16.6	11.6
Couple white men headed without children	3.2	3.6	3.4	2.7
White older adult or couple without aggregates	1.8	2.5	2.9	2.8
White older adult or couple with aggregates	4.8	4.7	4.7	4.4
Black older adult or couple without aggregates	0.8	1.7	2.0	2.0
Black older adult or couple with aggregates	4.7	5.4	5.8	5.4
Children (younger head)	88.0	81.9	80.1	76.8
Couple (younger head)	79.4	76.5	75.4	71.6
Women headed (younger head)	20.9	34.5	39.4	52.1
Black headed (all)	49.5	55.8	57.5	58.3
Old adult headed (all)	12.1	14.4	15.6	14.6

Source: *Brazilian National Household Sample Survey* (2004 ^13^ and 2013 ^14^), *Brazilian Household Budget Survey* (2017-2018 ^15^) and *Continuous Brazilian National Household Sample Survey* (2022 ^18^).

Some trends presented in the bottom panel are quite clear. As expected, population aging increased the percentage of individuals living in households headed by an old adult from 12.1% in 2004 to 14.6% in 2022.

Other expected general trends were confirmed due to changes commonly associated with the second demographic transition ^23^. Among the households headed by a young adult, the percentage of individuals living in households with couples and with children decreased from 79.4% and 88% in 2004 to 71.6% and 76.8% in 2022, respectively. Conversely, households headed by women increased remarkably: 20.9% of the population lived in households headed by a woman in 2004, number that increased to 52.1% in 2022.

Following the recent enhanced racial awareness in Brazil ^24^ the percentage of self-declared mixed/blacks ^25^ increased, facts that might be linked. Among all households, 49.5% lived in households headed by a black person in 2004, number that increased to 58.3% in 2022.

As for the percentage of individuals living in each of the 20 arrangements, those headed by an old adult showed little statistical differences between household head skin color; however, those headed by blacks slightly increased compared with those headed by whites. Moreover, most individuals in these households lived with children and/or aggregates, but households without them increased in the period.

Among households headed by young adults, two types (“traditional” household) were common in 2004 and decreased their participation remarkably: households with couples and children headed by white or black men. Both summed 60.2% in 2004 among all households and dropped to 27.9% in 2022. These were partially replaced by households with couples and children headed by white or black women, which increased from 3.3% in 2004 to 23.1% in 2022. Households headed by black women with children were significant, showing an increasing trend. All other arrangements were less frequent. Some showed increasing trends, all without children (black women headed without children, couple black women headed without children, black men headed without children, white women headed without children, couple white women headed without children and white men headed without children). One arrangement showed a decreasing trend (white women headed with children) and the remaining were somewhat stable (black men headed with children, couple black men headed without children, white men headed with children and couple white men headed without children).

Table 2 shows the evolution of food security and severe food insecurity in Brazil by household arrangements and characteristics in two panels. Last line of the upper panel shows the percentage evolution of all individuals facing these food insecurity levels between 2004 and 2018. Food security increased whereas severe food insecurity decreased between 2004 and 2013 and this trend reversed afterwards. All living arrangements followed the Brazilian general trend.

**Table 2:** Percentage of households food secure or with severe food insecurity by type and by characteristic between 2004 and 2018.

Characteristics	Food secure (%)			Severe food insecurity (%)		
	2004	2013	2018	2004	2013	2018
Black women with children	37.4	57.4	37.4	15.4	6.4	10.7
Black women without children	56.6	70.5	52.5	4.7	2.5	8.3
Couple black women headed with children	42.5	61.5	43.4	13.2	4.8	7.2
Couple black women headed without children	49.9	71.2	55.5	4.3	1.6	8.0
Black men with children	48.3	69.9	46.4	12.4	4.2	8.8
Black men without children	64.3	78.1	60.0	1.7	0.6	8.2
Couple black men headed with children	47.5	66.8	51.2	11.0	4.1	5.2
Couple black men headed without children	63.6	73.8	62.0	1.9	0.8	5.3
White women with children	60.3	75.5	58.4	6.0	2.7	6.3
White women without children	77.6	85.0	69.7	1.2	0.4	4.3
Couple white women headed with children	63.5	80.1	63.4	5.4	1.6	3.2
Couple white women headed without children	75.3	86.8	73.6	0.1	0.1	1.4
White men with children	67.4	83.5	74.9	3.2	1.5	2.6
White men without children	80.9	88.3	80.0	0.3	0.2	4.2
Couple white men headed with children	72.0	82.7	70.6	3.7	1.3	2.0
Couple white men headed without children	82.0	88.7	86.6	0.2	0.3	2.1
White older adult or couple without aggregates	85.2	91.5	72.8	0.0	0.0	1.3
White older adult or couple with aggregates	73.0	85.8	72.8	2.4	0.9	1.9
Black older adult or couple without aggregates	71.5	79.1	69.6	0.0	0.0	3.7
Black older adult or couple with aggregates	49.2	67.6	55.9	7.5	2.4	5.2
Total	60.0	73.8	59.1	6.8	2.7	4.9
Mean black difference	-20.6	-15.2	-19.7	5.0	1.8	4.1
Mean children difference	-13.9	-8.1	-11.1	7.0	2.5	0.6
Mean female difference	-7.9	-5.5	-9.0	2.0	0.9	1.4
Mean couple difference	-0.4	-0.4	-2.7	0.6	0.5	2.4

Source: *Brazilian National Household Sample Survey* (2004 ^13^ and 2013 ^14^), *Brazilian Household Budget Survey* (2017-2018 ^15^).

All households with food security values below the mean in 2004 were headed by a black person. Only households headed by men without children with or without a couple, and headed by an old black person without aggregates, had food security values above the mean. Households headed by single black women with children had the lowest food security values and the highest severe food insecurity means. The same arrangement but with a couple came next, followed by the same two types headed by men, and by households headed by old blacks with aggregates. In other words, food insecurity particularly touched black headed households with children and/or aggregates. Conversely, households with the highest food security values and lowest severe food insecurity means were all headed by whites without children, with or without a couple. Among those with the lowest food insecurity levels, only one arrangement had children: households headed by white men with a couple, a “traditional” type that is shrinking its participation.

Regarding households headed by older adults, aside ethnicity, differences between households with or without aggregates were significant. Note the lower food insecurity levels for households without aggregates, especially for 2004 and 2013.

The lower panel shows the mean differences for similar household types headed by black or white (black difference), with or without children (children difference), headed by women or men (female difference), and without or with a couple (no couple difference). As expected, all results for food security were negative and all for severe food insecurity, positive.

In 2013, period of greater food security, values were closer to zero for all comparisons, excepting the children difference for severe food insecurity. That is, the values for food security and severe food insecurity homogenized among household types in times of lower food insecurity levels and higher food security levels, but for this difference.

Results for 2004 and 2018 show that a household headed by a black when compared with a similar arrangement headed by a white had a nearly 20% difference for food security (40% against 60%), and a -5% difference for severe food insecurity (12% against 7%). This provided the greatest difference among the household characteristics.

Being child-free or not came next for food security. Households with children had an approximately 12% difference for food security; however, differences for severe food insecurity were greater in 2004 and minimal in 2018. In other words, having children in the household became nearly insignificant to determine severe food insecurity in 2018, but continued to be a negative factor to achieve food security.

Households headed by women showed an approximately 8.5% difference for food security and a 1.7% difference for severe food insecurity. Finally, the comparison between similar households with or without a couple showed the smallest differences, although they were greater in 2018.

### Econometric models: analysis per year

Table 3 summarizes the multinomial logistic model results for each year separately. The dependent variable has three categories, with the first being the standard for comparisons (secure/mild food insecurity; moderate food insecurity; severe food insecurity). Controls for location were included in the models, but the results are not shown.

**Table 3:** Results for multinomial logistic models between 2004 and 2018.

Variables	Moderate coefficients (SD)			Severe coefficients (SD)		
	2004	2013	2018	2004	2013	2018
Household arrangements and characteristics						
Black women with children			Reference			
Black women without children	-0.317 * (0.0755)	-0.0957 (0.0868)	-0.232 * (0.0861)	-1.783 * (0.153)	-1.159 * (0.168)	-0.117 (0.0992)
Couple black women headed with children	-0.170 ** (0.0769)	-0.291 * (0.0691)	-0.182 * (0.0667)	-0.270 * (0.0924)	-0.341 * (0.0891)	-0.329 * (0.0832)
Couple black women headed without children	-0.254 (0.160)	-0.191 (0.118)	0.0133 (0.100)	-1.666 * (0.340)	-1.441 * (0.268)	0.0894 (0.119)
Black men with children	-0.399 * (0.111)	-0.429 * (0.140)	-0.687 * (0.163)	-0.462 * (0.130)	-0.591 * (0.183)	-0.477 * (0.174)
Black men without children	-0.689 * (0.0678)	-0.330 * (0.0804)	-0.533 * (0.0841)	-3.545 * (0.223)	-3.497 * (0.343)	-0.267 * (0.0920)
Couple black men headed with children	-0.405 * (0.0385)	-0.541 * (0.0534)	-0.441 * (0.0570)	-0.624 * (0.0461)	-0.575 * (0.0691)	-0.740 * (0.0721)
Couple black men headed without children	-0.737 * (0.0640)	-0.579 * (0.0789)	-0.282 * (0.0756)	-2.448 * (0.142)	-2.151 * (0.191)	-0.588 * (0.0986)
White women with children	-0.263 * (0.0566)	-0.350 * (0.0882)	-0.00194 (0.0868)	-0.454 * (0.0737)	-0.331 * (0.115)	-0.0782 (0.109)
White women without children	-0.473 * (0.0904)	-0.297 ** (0.122)	-0.286 ** (0.116)	-2.437 * (0.260)	-2.060 * (0.347)	-0.373 * (0.143)
Couple white women headed with children	-0.424 * (0.107)	-0.487 * (0.106)	-0.234 ** (0.0999)	-0.647 * (0.144)	-0.815 * (0.158)	-0.553 * (0.140)
Couple white women headed without children	-0.599 * (0.229)	-0.895 * (0.224)	-0.247 * (0.149)	-3.328 * (1.015)	-3.387 * (1.004)	-0.962 * (0.256)
White men with children	-0.412 * (0.153)	-1.009 * (0.300)	-0.631 ** (0.260)	-1.214 * (0.258)	-1.075 * (0.399)	-0.607 ** (0.308)
White men without children	-0.947 * (0.0941)	-0.617 * (0.129)	-0.743 * (0.131)	-4.744 * (0.583)	-3.175 * (0.509)	-0.557 * (0.143)
Couple white men headed with children	-0.703 * (0.0440)	-0.950 * (0.0740)	-0.717 * (0.0754)	-1.011 * (0.0564)	-0.912 * (0.0965)	-1.164 * (0.107)
Couple white men headed without children	-1.122 * (0.0854)	-0.811 * (0.118)	-0.537 * (0.107)	-3.423 * (0.296)	-2.909 * (0.414)	-0.781 * (0.146)
White older adult without aggregates	-1.637 * (0.107)	-1.257 * (0.130)	-1.307 * (0.122)	-19.15 (790.1)	-18.48 (855.2)	-1.498 * (0.153)
White older adult with aggregates	-0.959 * (0.0744)	-1.217 * (0.123)	-0.569 * (0.103)	-1.525 * (0.117)	-1.781 * (0.207)	-1.055 * (0.152)
Black older adult without aggregates	-1.066 * (0.0865)	-0.843 * (0.0970)	-0.843 * (0.0887)	-19.73 (979.7)	-19.06 (927.9)	-1.261 * (0.116)
Black older adult with aggregates	-0.479 * (0.0563)	-0.618 * (0.0766)	-0.370 * (0.0748)	-0.990 * (0.0768)	-1.103 * (0.116)	-0.732 * (0.0981)
Formal education						
Less than half of primary education			Reference			
More than half to incomplete primary education	-0.296 * (0.0268)	-0.289 * (0.0397)	-0.305 * (0.0411)	-0.425 * (0.0371)	-0.488 * (0.0589)	-0.411 * (0.0500)
Incomplete secondary education	-0.609 * (0.0386)	-0.572 * (0.0507)	-0.395 * (0.0515)	-0.765 * (0.0564)	-0.835 * (0.0766)	-0.692 * (0.0661)
Complete secondary education	-0.946 * (0.0426)	-1.019 * (0.0532)	-0.593 * (0.0478)	-1.330 * (0.0708)	-1.338 * (0.0848)	-1.031 * (0.0637)
Tertiary education	-1.492 * (0.0798)	-1.607 * (0.0916)	-0.841 * (0.0671)	-1.707 * (0.142)	-1.817 * (0.157)	-1.339 * (0.0983)
Brick walls	-0.137 * (0.0368)	-0.241 * (0.0530)	0.0182 (0.0589)	-0.189 * (0.0492)	-0.353 * (0.0744)	-0.0434 (0.0695)
Sanitation	-0.120 * (0.0278)	-0.297 * (0.0383)	-0.127 * (0.0386)	-0.179 * (0.0389)	-0.369 * (0.0582)	-0.160 * (0.0501)
Waste collection	-0.0785 *** (0.0417)	-0.127 *** (0.0650)	0.0202 (0.0582)	-0.0891 (0.0549)	-0.478 * (0.0955)	-0.0662 (0.0737)
Television	-0.333 * (0.0319)	-0.371 * (0.0674)	-0.130 * (0.0401)	-0.506 * (0.0420)	-0.661 * (0.0983)	-0.230 * (0.0517)
Refrigerator	-0.322 * (0.0320)	-0.382 * (0.0639)	0.0351 (0.0678)	-0.524 * (0.0416)	-0.459 * (0.0926)	-0.384 * (0.0743)
Dweller density	0.805 * (0.0231)	0.895 * (0.0361)	1.154 * (0.0457)	1.115 * (0.0256)	1.170 * (0.0408)	1.399 * (0.0509)
Household income	-0.409 * (0.00833)	-0.229 * (0.00804)	-0.484 * (0.0190)	-0.486 * (0.0109)	-0.286 * (0.0121)	-0.636 * (0.0239)
Constant	0.841 * (0.123)	-1.046 * (0.181)	1.660 * (0.221)	0.956 * (0.170)	-0.880 * (0.266)	2.761 * (0.294)
Observations	109,415	109,403	57,111	109,415	109,403	57,111

SD: standard error.

Source: *Brazilian National Household Sample Survey* (2004 ^13^ and 2013 ^14^), *Brazilian Household Budget Surve*y (2017-2018 ^15^).

Note: controls for urban areas and states. Variance inflation factor (VIF) tests did not show multicollinearity issues.

* p < 0.01;

** p < 0.05;

*** p < 0.1.

Regarding the categorical variable for household arrangements and characteristics, the arrangement with the highest food insecurity level - black women headed without a couple and with children - was used as reference. Results showed that most coefficients were negative and significant, indicating that the remaining living arrangements had a smaller likelihood of having moderate or severe food insecurity, even after controlling for the other variables. That is, formal schooling, household characteristics, household income and location could only partially explain the differences in food insecurity observed in Table 2. Only two household types had more than one non-significant coefficient: couple black women headed couple without children (four coefficients) and black women headed without children (two coefficients). In other words, differences between these arrangements and the reference were small and could be explained by model controls. No positive and significant coefficient was observed. Coefficients for households headed by older adults without aggregates for severe food insecurity in 2004 and 2013 should not be considered, as these categories had no household with severe food insecurity.

Comparing the coefficients for moderate food insecurity with those for severe food insecurity, the latter were smaller (larger modulus), that is, households headed by black women with children were particular touched by severe food insecurity. However, differences were smaller in 2018 due to more widespread cash transfers policies.

General trends for formal schooling are quite clear: all coefficients are negative and significant. Household heads with higher levels of formal schooling were less likely to be food insecure, as expected. Comparing moderate with severe food insecurity, education seems to have greater effect on the latter. That is, households headed by more educated individuals were particular more effective in overcoming severe food insecurity. By 2022, however, higher levels of education had smaller effects for both types of food insecurity. As higher levels of formal education became more widespread in Brazil ^26^, its effects on food insecurity became smaller.

As for the other socioeconomic variables, we observed some general trends for both types of food insecurity. Those who lived in households without brick walls, sanitation, waste collection, television and refrigerator were more likely to be food insecure. Moreover, poorer households and those with greater dweller density were more likely to face both types of food insecurity. All these variables are positively correlated and indicate the existence of severe deprivation in a multidimensional perspective ^10^, including food insecurity.

### Estimation of moderate/severe food insecurity before and during the COVID-19 pandemic by household arrangements and characteristics

Table 3 control variables correlate with food insecurity levels in the long term, but show poor associations with its short-term temporal evolution. Population distribution among states or between urban and rural areas varied in the last decades, but had a negligible impact on food insecurity levels. As in the last two decades individuals became increasingly more educated and dwelling conditions improved, the trend observed for these explanatory variables would be of decreasing food insecurity.

This section used PNAD and Continuous PNAD data, which allowed us to incorporate other labor market-related variables, more closely linked to food insecurity increases during the pandemic, as explanatory variables ^9^. The logistic model had a dummy as dependent variable (secure/mild food insecurity; moderate/severe food insecurity) and was estimated using 2013 PNAD.

Table 4 shows the logistic model results for living arrangements, labor market outcomes, non-labor market funds and some other controls. Control findings are not shown, as they were already discussed in Table 3 and the results were similar and robust. Results of the interactions are not shown for brevity.

**Table 4:** Results for logistic models in 2013.

Household arrangement and head’s characteristics	Coefficients (SD)
Black women with children	Reference
Black women without children	-0.632 * (0.0457)
Couple black women headed with children	0.0962 * (0.0363)
Couple black women headed without children	-0.396 * (0.0603)
Black men with children	-0.244 * (0.0695)
Black men without children	-0.695 * (0.0436)
Couple black men headed with children	-0.198 * (0.0290)
Couple black men headed without children	-0.520 * (0.0387)
White women with children	-0.484 * (0.0472)
White women without children	-1.199 * (0.0604)
Couple white women headed with children	-0.445 * (0.0529)
Couple white women headed without children	-1.033 * (0.0828)
White men with children	-0.872 * (0.113)
White men without children	-1.210 * (0.0661)
Couple white men headed with children	-0.659 * (0.0451)
Couple white men headed without children	-1.188 * (0.0576)
White older adult without aggregates	-1.635 * (0.112)
White older adult with aggregates	-0.764 * (0.109)
Black older adult without aggregates	-0.860 * (0.107)
Black older adult with aggregates	-0.0136 (0.105)
Unemployed	0.765 * (0.0426)
Resources other than labor	0.429 * (0.0175)
Regional underemployment	0.0591 * (0.00138)
Controls for labor market income and workload	Yes
Control for urban area	Yes
Interactions	Yes
Observations	110,840

SD: standard errors.

Notes: (1) all models include the categorical variable for state as control; (2) model 4 includes interactions of selected variables; (3) Variance inflation factor (VIF) tests did not show multicollinearity issues.

* p < 0.01.

Even after incorporating all these variables into the models and interactions, most differences between household arrangements were still significant. Households with an unemployed head were more likely to suffer moderate/severe food insecurity, as expected. Households that received non-labor market funds were more likely to face food insecurity, but the main causality here might be that individuals with high levels of deprivation are more prone to procure resources from other sources. Households in states with a greater percentage of individuals outside the labor market, unemployed or underemployed were more likely to face food insecurity.

Table 5 summarizes the moderate/severe food insecurity estimates after great Brazilian recession and during the COVID-19 pandemic based on three scenarios. In the first scenario, which does not include the emergency aid nor pandemic idiosyncrasies, the moderate/severe food insecurity values were adjusted to the actual moderate/severe food insecurity level observed in 2018, 15.9% for households or 16.5% for individuals. Moderate/severe food insecurity increased between 2018 and 2020, in part due to the effects of the pandemic on labor market outcomes. The 23.5% mean in 2020 was slightly above those observed in VIGISAN I ^8^ for the same year, 20.5% for households and 21.6% for individuals. Mostly based on labor market outcomes, the estimates showed much lower values in 2021 (20.7%) than that observed by VIGISAN II ^8^ (30.7% or 32.6%, respectively). Labor market outcomes in 2021 were better than in 2020, but the emergency aid became less widespread in 2021. In 2022, moderate/severe food insecurity estimates were still higher than in 2018. This general trend was also observed for household types.

**Table 5:** Percentage of individuals in moderate or severe food insecurity by household arrangement and characteristics between 2018 and 2022 in different scenarios.

Household arrangements and characteristics	Scenario 1 (%)				Scenario 2 (%)			Scenario 3 (%)	
	2018	2020	2021	2022	2020	2021	2020	2021	2021
Emergency aid included	No	No	No	No	Yes	Yes	No	Yes	No
Black women with children	24.1	31.9	30.9	29.8	28.2	29.3	32.0	47.0	50.6
Black women without children	16.4	23.8	19.3	22.1	21.1	16.5	23.9	28.1	32.9
Couple black women headed with children	22.2	30.6	28.2	27.8	30.0	27.9	30.9	45.8	47.1
Couple black women headed without children	18.4	25.4	21.3	21.9	23.0	20.3	25.5	33.7	36.0
Black men with children	19.5	30.5	26.3	26.3	28.6	25.2	30.5	42.4	44.0
Black men without children	14.2	21.8	18.5	17.2	18.9	15.9	22.0	29.3	31.6
Couple black men headed with children	20.8	27.9	25.2	23.8	27.1	24.5	28.0	41.4	42.4
Couple black men headed without children	16.4	25.9	20.7	20.6	23.8	19.1	26.0	33.4	35.4
White women with children	15.9	23.0	20.0	20.6	19.5	18.3	23.2	30.0	32.3
White women without children	8.0	15.4	13.7	12.9	11.8	12.1	15.4	17.8	21.0
Couple white women headed with children	13.8	19.5	15.7	17.3	19.0	15.4	19.5	25.9	26.9
Couple white women headed without children	8.5	14.0	13.0	11.6	12.7	12.2	14.1	18.4	19.8
White men with children	11.7	21.8	12.6	13.2	20.0	11.1	21.9	21.3	21.9
White men without children	6.8	12.4	10.0	10.5	10.8	8.9	12.5	15.9	17.8
Couple white men headed with children	11.4	19.5	14.6	15.0	18.8	14.4	19.6	24.2	24.7
Couple white men headed without children	6.6	11.0	11.4	10.7	9.7	10.5	11.1	17.5	18.6
White older adult without aggregates	6.5	9.1	9.1	8.0	4.7	5.9	9.1	9.2	14.3
White older adult with aggregates	8.8	14.8	11.7	12.2	12.0	9.9	14.9	16.3	20.3
Black older adult without aggregates	14.4	20.2	16.9	18.7	13.3	11.0	20.2	19.1	28.9
Black older adult with aggregates	18.9	26.0	23.6	22.2	22.5	20.6	26.1	35.3	39.4
Total	16.5	23.5	20.7	20.4	21.7	19.5	23.6	32.6	34.6

Source: *Brazilian National Household Sample Survey* (2004 ^13^), and *Continuous Brazilian National Household Sample Survey* (2018 ^16^, 2020-2021 ^17^, and 2022 ^18^).

The second scenario included the emergency aid and indirectly incorporated idiosyncrasies of the first pandemic year, and the moderate/severe food insecurity values were adjusted to the actual levels observed in 2020 (21.7% for individuals). Comparison of this column with the 2021 results indicates that moderate/severe food insecurity I levels would be lower in this year if the evolution of labor market variables and emergency aid determined these levels, which is contrary to the findings. Comparison between the two columns with 2020 data estimated the effects of the emergency aid on food insecurity: considering it, 21.7% of all individuals would live in households with moderate/severe food insecurity; without it, this number would increase to 23.6%.

In the third scenario, which includes the emergency aid and indirectly incorporated idiosyncrasies of the second pandemic year, moderate/severe food insecurity values were adjusted to the actual levels observed in 2021 (32.6% for individuals). When comparing the two columns with 2021 data, we observe that 15.5% of households receiving the emergency aid would be moderate/severe food insecurity, and 16.6% would be moderate/severe food insecurity without it.

All household types suffered with the pandemic, but those headed by an older adult without aggregates showed the smallest variations, whereas those headed by Whites presented the lowest values. Households headed by single black women with children had the highest value in most estimates, but the 2020 emergency aid was particularly helpful for this arrangement.

## Discussion

Between 2004 and 2022, Brazil’s household composition distribution evolved following changes commonly associated with the second demographic transition, which transformed household formation patterns ^23^: female labor-force participation increased; age at first marriage increased, with a postponement of fertility; cohabitation and divorce rates increased; individual autonomy and expressive values tied to self-actualization became more widespread; and agreements about gender roles within marriage became less rigid. Due to these cultural changes, households headed by child-free single women relatively increased in Brazil. 

Moreover, this evolution also followed the recent emergence of racial awareness in Brazil which promoted further and deeper discussions about race at an unseen level ^24^. Ethnic and racial identities are built based on historical and cultural perspectives, as subjects recognize themselves as members of particular social groups. Consequently, the proportion of self-declared black/mixed individuals increased in Brazil and households headed by blacks relatively increased in the period.

Households headed by blacks and single women with children had a greater propensity of food insecurity, as also observed by other authors ^6^ ^,^ ^7^ ^,^ ^8^. Ethnic and gender differences in household heads were quite persistent to determine food insecurity levels between 2004 and 2018. Presence of a couple or of children, probably due to the expansion of cash transfer polices ^27^, became less important. In other words, these policies decreased the importance of two people earning labor market income in the household, thus more effectively buffering children from food insecurity.

Similarly to this study, other authors ^28^ ^,^ ^29^ observed that black/mixed individuals faced higher levels of moderate/severe food insecurity. These authors emphasized that food insecurity is one of the many aspects related to marginalization due to race/skin color by structural racism, also pointing out that the intersection between sexism and racism poses greater obstacles to black women’s autonomy, decreasing their power of choice. Moreover, some studies ^30^ highlight the more precarious conditions of households headed by blacks in several other features besides food insecurity, such as schooling levels, household income and housing conditions.

Associated with these social marginalization aspects, other different factors influence the formation of households headed by blacks ^30^. Regarding interracial marriages in Brazil, mixed individuals have a greater chance of marrying whites than blacks, as marriages of physically similar types are more accepted. Thus, the distances separating whites, mixed and blacks in the marriage market does not resemble strictly socioeconomic gaps, as mixed individuals are much closer to blacks socioeconomically. Nonetheless, interracial marriages increased in Brazil between 1960 and 2000 ^31^.

Great Brazilian recession and the COVID-19 pandemic outbreak negatively impacted the economy ^2^ ^,^ ^3^ ^,^ ^4^ and food insecurity levels in Brazil ^6^ ^,^ ^7^ ^,^ ^8^ ^,^ ^9^. However, the pandemic had short-term idiosyncrasies on food insecurity that could not be explained by socioeconomic or demographic variables. Social isolation and diminished social and solidarity informal ties might have also impacted food insecurity.

## Conclusion

Analysis of household and individual data shows that food insecurity is highly correlated with human development and well-being indicators, such as poverty, access to drinking water and basic sanitation services ^1^, malnutrition, stunting, wasting and common childhood infectious diseases ^32^. Food deprivation and poor dietary quality during childhood influence children’s growth and their psycho-emotional, social, and cognitive development, hindering economic development and perpetuating social inequalities. Moreover, for both youth and adults, the positive impacts of better nutrition lead to direct gains in productivity arising from improved physical strength and indirect gains resulting from enhanced learning and cognitive development. Thus, great Brazilian recession and the COVID-19 pandemic may have long lasting and unforeseen negative consequences on human well-being due to increases in food insecurity in particular years.

Identifying the population groups most vulnerable to the deleterious effects of food insecurity is critical for designing, implementing, and effectively targeting successful public policy programs. As such, we identified the most vulnerable living arrangements.

Actions to combat food insecurity should be based on multisectoral collaboration involving agriculture, health care, water and sanitation services, and educational systems. Inclusive policies with a multisectoral design and aimed at vulnerable people should be implemented, integrating food security and nutrition into poverty focused policies, as synergies help accelerate social goals.
